# Current insights on gut microbiome and chronic urticaria: progress in the pathogenesis and opportunities for novel therapeutic approaches

**DOI:** 10.1080/19490976.2024.2382774

**Published:** 2024-07-30

**Authors:** Rui Cai, Changhan Zhou, Ruisi Tang, Yuanling Meng, Jumei Zeng, Yuqing Li, Xiang Wen

**Affiliations:** a West China School of Medicine, West China Hospital, Sichuan University, Chengdu, Sichuan, China; b State Key Laboratory of Oral Diseases, National Clinical Research Center for Oral Diseases, West China Hospital of Stomatology, Sichuan University, Chengdu, Sichuan, China; c West China School of Public Health and West China Fourth Hospital, Sichuan University, Chengdu, Sichuan, China; d Department of Dermatology, West China Hospital, Sichuan University, Chengdu, Sichuan, China; e Laboratory of Dermatology, Clinical Institute of Inflammation and Immunology, Frontiers Science Center for Disease-related Molecular Network, West China Hospital, Sichuan University, Chengdu, Sichuan, China

**Keywords:** Gut microbiome, chronic urticaria, dysbiosis, inflammation, infection, microbiota therapy

## Abstract

Chronic urticaria (CU) is a prevalent skin disorder greatly impacting the patients’ life quality, in which immune dysregulation mediated by gut microbiome plays a significant role. Several studies have found the gut dysbiosis exists in patients with CU. In addition, infection may also be one of the causes of CU. The primary treatment currently used for CU is the second-generation non-sedating H1-antihistamines (nsAH). However, there are some limitations in current therapies. Based on the latest evidence, this review provides an updated overview of how the gut dysbiosis influences CU development, explores potential therapeutic approaches based on the gut microbiota and summarizes the interaction between gut microbiota and current treatment.

## 1. Introduction

Urticaria is an immune-related skin disorder that affects up to 20% of individuals over their lifetime. It can present as a pruritic rash, angioedema, or both.^1,2^ Urticaria, also known as hives, can be categorized into two main types: acute urticaria (AU) and chronic urticaria (CU). AU is mostly a self-limiting disease with a duration of less than 6 weeks, mostly related to infections, drugs or certain foods.^1^ CU is characterized by spontaneous or inducible occurrence, lasting more than 6 weeks without a clear etiology,^1^ including chronic spontaneous urticaria (CSU) and chronic inducible urticaria. It greatly impacts the quality of life of patients and imposes a substantial burden on them.^3^

The development of CU is considered to be closely related to the immune system.^4^ Relevant studies have shown that CU lesions have a large infiltration of immune cells,^5^ such as mast cells,^6^ eosinophils^7^ and basophils.^8^ The activation of mast cells is a key factor in the development of CU, as it triggers the release of histamine, resulting in the characteristic symptoms of the disease.^6^ The researchers hypothesized that eosinophils in the blood are recruited to the skin during the active phase of CU, which is related to the interaction between eosinophils and mast cells through various mediators such as cytokines.^7^ During the active phase of CU, basophils in the blood are also recruited to the skin, influencing the pattern of IgE receptor-mediated mast cell degranulation.^8^ The interactions between mast cells and different immune cells are crucial in shaping the local microenvironment.^4^

The current primary treatment for CU is the second-generation non-sedating H1-antihistamines (nsAH).^1^ Evidence from randomized controlled trials strongly advocates for the use of these antihistamines.^1,9,10^ In cases where CU is inadequately controlled, adjunct therapies such as second-generation H1-antihistamines, leukotriene drugs, and short-term oral glucocorticoids may be considered.^2^ If the above treatments are ineffective, omalizumab may be considered. There is compelling evidence to advocate for the use of cyclosporine after ineffective treatment with omalizumab.^11^ However, current treatments have limited effectiveness in some patients with CU.^2^ In recent years, several innovative targeted therapeutic options such as dupilumab^12,13^ and Bruton’s tyrosine kinase inhibitors,^12,13^ are currently undergoing clinical trials for the treatment of CU.

Recent studies have shown that an imbalance in the gut microbiota is associated with the development of certain skin diseases.^14,15^ Clinical studies have demonstrated alterations in the gut microbiota of CU patients, indicating a potential role of the gut microbiota in the development of CU.^16–21^

In this review, we provide a comprehensive overview of the currently available literature on gut dysbiosis in patients with CU and explore potential treatment strategies based on the gut microbiota.

## 2. Gut microbiome and chronic urticaria

### 2.1. Gut microbiome in chronic urticaria

It is universally acknowledged that CSU is mainly an immune-mediated inflammatory disease. Both Type I and Type II autoimmunity, specifically the presence of IgE antibodies to autoallergens and IgG autoantibodies to IgE or its high-affinity receptor (FcεRI), are thought to play a role in the development and progression of CSU.^22^

**Figure 1.**
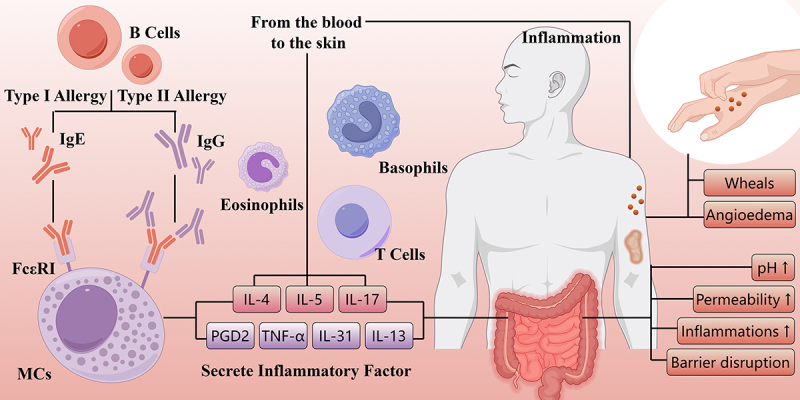
The manifestations and pathophysiology of chronic urticaria.

The activation, degranulation, and mediator release from cutaneous mast cells are crucial to the pathogenicity of CSU.^23^ The symptoms of CSU primarily result from the release of histamine, along with other mediators including prostaglandin D2 (PGD2), tumor necrosis factor (TNF) and various interleukin (IL), such as IL-4, IL-5, IL-13, IL-17, and IL-31, which can impact resident skin cells and other recruited target cells like T cells, eosinophils, and basophils. These recruited cells migrate from the blood into the skin in response to chemotactic factors, such as IL-5, complement 3a (C3a), complement 5a (C5a), TNF, IL-17 and so on, released by mast cells, activated endothelial cells, T helper (Th) 2 cells and other cells.^24^ (Figure 1) A healthy gut is essential for overall health. In most scientific literature, the term “gut microbiota” or “gut flora” specifically refers to the bacteria that reside in the gastrointestinal tract of humans and other animals. Studies have indicated that the imbalance in the gut microbiota may contribute to skin diseases. Dysbiosis is linked to skin diseases like psoriasis, atopic dermatitis, and acne through inflammation and immune system disruption.^25^ CU, an immune-mediated skin disease, may also be influenced by gut flora.^4^ CSU is the primary focus of relevant studies among the different subtypes of CU.

Both IgE-mediated type I and IgG-mediated type II allergies are the main pathophysiological mechanisms of CU, through which the MCs are activated and then release various inflammatory factors. Eosinophils, T cells and basophils can be recruited from the blood to the skin in response to these factors. Lower PH, higher permeability, elevated level of inflammation and the barrier disruption can be discovered in the intestine of CU patients, whose skins show wheals and angioedema.

CU, chronic urticaria; MCs, mast cells; PDG2, prostaglandin D2; TNF-ɑ, tumor necrosis factor-ɑ; IL, interleukin; FcεRI, high-affinity IgE receptor (By Figdraw).

Recent studies have examined the gut flora of patients with CSU, highlighting notable disparities in microbial composition between CSU patients and healthy controls (HCs). Inconsistencies in findings may stem from different CSU’s subtypes and varied diagnostic techniques. The majority of research employed 16S rRNA sequencing to compare species and genera in HC and CSU groups. In some studies, quantitative polymerase chain reaction (PCR) or real-time PCR were used to identify species-specific variations (Table 1). Additionally, metabolomic analyses have also been instrumental in investigating metabolite alterations in CSU patients, offering insights into the disease’s pathogenesis.^26,27^

**Table 1.** A summary of current evidence on the association between gut microbiome composition and chronic urticaria (mainly CSU).

Authors	Observation group (sample size)	Control group (sample size)	Detection technology	Increased gut microbiota in patients	Decreased gut microbiota in patients	Increased metabolites in *vivo*	Decreased metabolites in *vivo*	References	alpha diversity compared with control group
Zhu et.al (2023)	CSU (Stool:26; Blood:29)	HC (Stool:26; Blood:38)	metagenomics sequencing and short-chain fatty acids metabolomics	**Family** Enterobacteriaceae, Peptostreptococcaceae **Genus** Klebsiella **Species** Klebsiella pneumoniae, Bacteroides stercoris, Escherichia coli	**Family** Rikenellaceae **Genus** Alistipes **Species** Roseburia hominis, Clostridium leptum, Bacteroides, Ruminococcus obeum	LPS	SCFAs	Gut microbiota facilitate chronic spontaneous urticaria	Low
Wang et.al (2020)	CSU (10)	HC (10)	16S rRNA gene sequencing and untargeted metabolomic analyses	**Family** Enterobacteriacea	**Phylum** Firmicutes; **Genus** Bacteroides, Faecalibacterium, Bifidobacterium, Lactobacillus, Ruminococcaceae		docosahexaenoic acid, arachidonic acid, glutamate succinic acid	Gut Microbiome and Serum Metabolome Analyses Identify Unsaturated Fatty Acids and Butanoate Metabolism Induced by Gut Microbiota in Patients with Chronic Spontaneous Urticaria	Low
Wang et.al (2021）	CSU (39)	HC (40)	16S rRNA gene sequencing	**Phylum** Firmicutes, **Genus** Faecalibacterium, Roseburia, Lachnospira, Gemmiger, Prevotella, Bifidobacterium	**Phylum** Proteobacteria **Genus** Blautia, Ruminococcus, Oscillospira, Megamonas, Dialister, Bacteroides, Parabacteroides, Alistipes, Sutterella	a-mangostin, glycyrrhizic acid	3-indolepropionic acid, xanthine, isobutyric acid	Abnormalities in Gut Microbiota and Metabolism in Patients With Chronic Spontaneous Urticaria	low
Lu et.al (2019)	CU (10)	HC (10)	16S rRNA sequencing	**Phylum** Proteobacteria, Actinobacteria **Order** Enterobacteriales, Lactobacillales, Pseudomonadales **Genus** Veillonella, Sutterella, Streptococcus, Clostridium, Escherichia **Species** E. coli	**Phylum** Bacteroidetes **Genus** Faecalibacterium, Prevotella, Lachnobacterium **Species** Faecalibacterium prausnitzii, Prevotella copri, Bacteroides fragilis, Bacteroides plebeius			Altered Gut Microbiota Diversity and Composition in Chronic Urticaria	low
Nabizadeh et.al (2017)	CU (20)	HC (20)	Real-time PCR	**Family** Enterobacteriaceae	**Genus**: Akkermansia, muciniphila, Clostridium leptum, Faecalibacterium prausnitzii			Association of altered gut microbiota composition with chronic urticaria	low
Liu et.al (2021)	CSD (25)	HC (25)	16S ribosomal DNA sequencing, QPCR	**Phylum** Fusobacteria **Class** Gammaproteobacteria, Fusobacteria **Order** Enterobacteria, Fusobacteria **Family** Enterobacteriaceae, Fusobacteraceae, Peptostreptococcaceae, Streptococcaceae **Genus** Klebsiella	**Phylum** Firmicutes **Class** Clostridia, Alphaproteobacterial, Deltaproteobacteria **Order** Clostridiales, Rhodospirillales, Caulobacterales, Desulfovibrionales **Family** Ruminococceae, Rikenellaceae, Muribaculaceae, Christensenellaceae, Caulobacteraceae **Genus/species** Subdoligranulum, Ruminococcus bromii			Biomarkers of Gut Microbiota in Chronic Spontaneous Urticaria and Symptomatic Dermographism	low
Rezazadeh et.al (2018)	CU (20)	HC (20)	PCR, Real-time PCR		**Genus** Lactobacillus, Bifidobacterium			The protective effect of Lactobacillus and Bifidobacterium as the gut microbiota members against chronic urticaria	
Luo et.al (2023)	CSU (15)	HC (15)	16S rRNA gene sequencing	**Phylum** Firmicutes **Genus** Faecalibacterium, Roseburia, Prevotella, Dialister, Coprococcus, Gemmiger, Oscillospira, Lachnospira	**Phylum** Bacteroidetes, Proteobacteria **Genus** Bacteroides, unidentified- Ruminococcus, Pseudomonas, Megamonas, Lactobacillus	purine and other nucleotide metabolites	Unsaturated fatty acids	Combined microbiome and metabolome analysis of gut microbiota and metabolite interactions in chronic spontaneous urticaria	high
Zhang et.al (2021)	CSU (20)	HC (20)	16s rRNA massive sequencing	**Phylum** Firmicutes, Bacteroidetes, Proteobacteria, Verrucomicrobia, Class Bacilli **Order** Enterobacterales **Family** Enterobacteriaceae	**Genus** Megamonas, Megasphaera, Dialister			Gut Microbiome Alterations and Functional Prediction in Chronic Spontaneous Urticaria Patients	No significant difference
Yüksekal et.al (2022)	CSU (20)	HC (20)	16S RNA sequencing, bioinformatic analysis	**Phylum** Bacteroidetes **Family** Lachnospiraceae, Ruminococcaceae, Clostridiaceae **Genus** Intestinibacter, Megasphaera, Sutterella	**Order** Clostridiales **Family** Bifidobacteriaceae, Lachnospiraceae, Ruminococcaceae, Veillonellaceae, Prevotellaceae, Coriobacteriaceae **Genus** Succinivibrio			Investigation of intestinal microbiome in chronic spontaneous urticaria patients	high
Ćesić et.al (2023)	CSU (22)	HC (23)	16S rRNA sequencing	**Genus** Bacteroides, Streptococcus, Agathobacter, Bifidobacterium, Lactobacillus	**Class** Clostridia **Family** Lachnospiraceae **Genus** Roseburia, Faecalibacterium, Ruminococcus, Lachnospira, Prevotella, Blautia, Coprococcus, Subdoligranulum, Eubacterium eligens			Association of Gut Lachnospiraceae and Chronic Spontaneous Urticaria	Low

CU: chronic urticaria; CSU: chronic spontaneous urticaria; HC: healthy control; SCFAs: short-chain fatty acids; LPS: lipopolysaccharide.

Alpha diversity of gut flora refers to the diversity of species in a single sample and can be measured using metrics such as Chao1 value, ACE value, and Shannon index.^28^ Beta diversity is a comparison of differences in diversity between samples.^19^ Studies have predominantly found lower alpha diversity in patients with CSU compared to HCs, along with a significant difference in the composition of gut flora between the two groups.^17,21,26,27,29^

Except bacteria, the parasite is also an essential component of gut microbiome.^30,31^ Currently, parasite infection (PI) is described as an underlying cause of CSU.^32^ There are a variety of studies concerning the prevalence of PI in CSU and possible mechanisms.

Although virus and fungi also belong to gut microbiome, there are few researches directly focusing on the role of intestinal virus and fungi in the pathogenesis of chronic urticaria. Retrieved studies and cases mainly reported patients infected with enterovirus were susceptible for acute urticaria.^33–35^ As regard to the fungi, food yeasts and increased rates of sensitization to *Candida albicans* may be contributors to CU, but specific mechanisms have not been clarified.^36,37^

#### 2.1.1. Increased levels of opportunistic pathogens in CU patients

*Enterobacteriaceae* family is one of the pro-inflammatory members of the gut microbiota.^38^ Nabizadeh et al. conducted the initial study to explore changes in the gut microbiome of patients with CU using real-time PCR.^20^ The results revealed that the *Enterobacteriaceae* family was more prevalent in the fecal samples of CU patients, aligning with findings from other studies.^17,19,26,27^ Lu et al. also observed a higher abundance of *Escherichia coli* and *Klebsiella spp*. in the CSU group.^39^ A recent study analyzed the composition of gut microbiome in patients with CSU using multi-omics analysis, which revealed that CSU patients have lower gut flora diversity but higher levels of *Klebsiella pneumoniae*.^27^ Liu et al. observed a positive correlation between *Clostridium disporicum* and low quality of life.^17^

At the phylum level, the gut microbiota composition in CSU patients is comparable to that of HCs, comprising *Firmicutes*, *Bacteroidetes*, *Proteobacteria*, *and Actinobacteria*.^29^ In the study by Zhang et al. significant differences were observed between the CSU and HC groups, including *Proteobacteria* at the phylum level, *Bacilli* at the class level, *Enterobacterales* at the order level, *Enterobacteriaceae* at the family level, and *Megamonas*, *Dialister*, and *Megasphaera* at the genus level.^19^ The study particularly emphasized a pronounced increase in the phylum *Proteobacteria* within the CSU group, a finding supported by Wang et al. and Lu et al.^29,39^

In addition to the aforementioned findings, Wang et al.‘s study identified higher levels of the genera *Lactobacillus*, *Turicibacter*, and *Lachnobacterium* in the CSU group.^29^ Shi et al. explored the causal relationship between gut flora and CSU using Mendelian randomization analysis. The study identified phylum *Verrucomicrobia*, genus *Defluviitaleaceae UCG011* and genus *Coprococcus 3* as potential risk factors for urticaria.^40^ *Helicobacter pylori* (HP), known to colonize the stomach and duodenum, can lead to persistent infections. A meta-analysis showed that patients with CU had a higher prevalence of HP infection than controls (odd ratio = 1.66, 95%CI:1.12–2.45; *p* = 0.01) and that HP-negative patients were more likely to experience spontaneous remission of symptoms than positive patients.^41^ Another study showed that the eradication of bacteria doubled the probability of clinical remission of CU.^42^ To explain these findings, researchers proposed one hypothesis suggesting that *H. pylori*‘s 21–35 kDa mixed protein components may stimulate the degranulation of human mast cells.^43^ In addition, some bacterial genes such as *cagA, vacA* and *nap* may encode specific proteins triggering an immune response that enhances pro-inflammatory pathways.^44^

#### 2.1.2. Decreased levels of beneficial bacteria in CU patients

*Bacteroidetes* is a predominant component of the intestinal flora. Genus *Bacteroides* is the primary producer of short-chain fatty acids (SCFAs), specifically acetic and propionic acids.^45^ Luo et al. discovered that the abundance of phylum *Bacteroidetes* was lower in CSU patients compared to the HCs, suggesting a possible protective role of *Bacteroidetes* against CSU.^46^ Research has shown that *Bacteroides* exerts a regulatory influence on human immunity, mainly by producing capsular polysaccharide A and SCFAs. Capsular polysaccharide A plays a role in maintaining and balancing immune system function, as well as in preventing bacterial and viral infections.^45^ Both acetate and propionate serve as potent anti-inflammatory agents, capable of suppressing the release of pro-inflammatory cytokines by neutrophils and macrophages.^47^

It is worth noting that *Firmicutes* and *Bacteroidetes* constituted 90% of the beneficial bacterial strains in the intestine.^48^ Phylum *Firmicutes* can break down insoluble dietary fiber to release nutrients and promote the proliferation of different bacterial species in the gut.^49^ Liu et al. and Wang et al. reported a decrease in the number of phylum *Firmicutes* in the CSU group.^17,26^ However, this conclusion has not yet reached a consensus.^19,29,46^

Moreover, Wang et al. found that genus *Phascolarctobacterium* was higher in the healthy group.^29^ And patients with chronic spontaneous urticaria and symptomatic dermographism (CSD) have significantly lower levels of *Subdoligranulum* and *Ruminococcus bromii*, which may have diagnostic values.^17^

Rezazadeh et al. discovered that the relative amounts of *Lactobacillus* and *Bifidobacterium* were significantly higher in fecal samples from controls compared to patients with CU, indicating a protective effect against CU.^35^ This may be attributed to their ability to induce regulatory T (Treg) cells.^36^ Ćesić et al. found reduced levels of *Lachnospiraceae* members responsible for SCFA production in the gut flora of patients with CSU.^50^ In the Mendelian Randomization analysis, order *Burkholderiales* and genus *Eubacterium xylanophilum* group were found to be potentially protective against urticaria.^40^

### 2.2. Influence of gut microbiome in chronic urticaria

**Figure 2.**
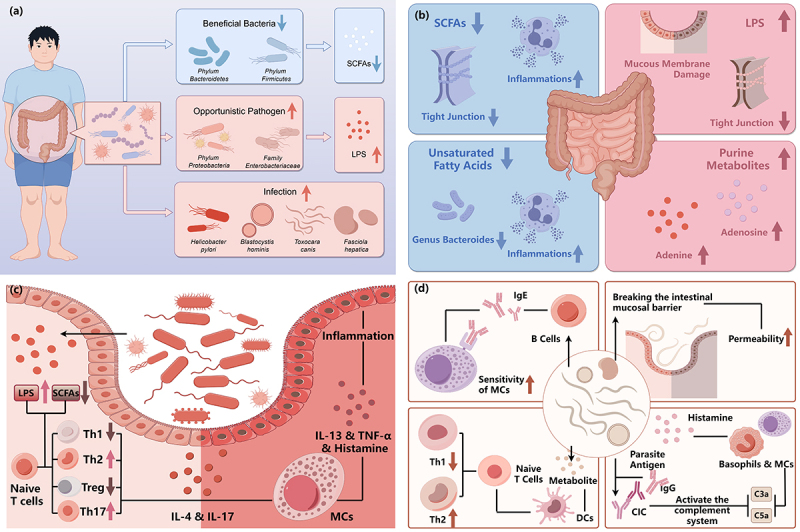
The alteration and influence of gut microbiome in chronic urticaria.

As mentioned above, visible changes happened in the gut microbiome of CSU patients. Mast cells are more likely to be activated in patients with CSU compared to the HCs, which may be related to a lower threshold for activation signals or reduced exposure to inhibitory signals.^51,52^ Lipopolysaccharide (LPS) and pro-inflammatory cytokines can make mast cells more susceptible to activation, whereas SCFAs can inhibit mast cells activation.^53^ These factors affecting mast cells’ activation can be produced by the gut flora. (Figure 2).

(a) Gut dysbiosis: decreased levels of SCFAs-producing pathogens, increased levels of opportunistic pathogens and relevant intestinal infections, including HP and parasite. (b) Changes of metabolites: the levels of SCFAs and unsaturated fatty acids are reduced in the intestine of CU patients, while those of LPS and purine metabolites are elevated. (c) Intensified inflammation in intestine: The upregulation of LPS and downregulation of SCFAs appear as a result of gut dysbiosis, contributing to the abnormal differentiation of naïve T cells. The levels of IL-4 and IL-17 are elevated due to more Th2 and Th17, and less Treg and Th1. MCs are activated to release some inflammatory mediators like IL-3, TNF-ɑ and histamine. (d) Roles of parasite infection: enhancing the sensitivity of MCs; breaking the mucosal barrier and amplify the intestinal permeability; producing metabolites and interact with DCs to influence the differentiation of naïve T cell; forming CIC (IgG and parasite antigen) and activate the complement system to promote the release of inflammatory mediators.

SCFAs, short chain fatty acids; HP, helicobacter pylori; CU, chronic urticaria; LPS, lipopolysaccharides; IL, interleukin; TNF-ɑ, tumor necrosis factor-ɑ; Th, T helper cells; Treg, regulatory T cells; MCs, mast cells; C3a, complement 3a; C5a, complement 5a; DCs, dendrite cells; CIC, immune complexes (By Figdraw).

#### 2.2.1. The disrupted immune regulatory function of SCFAs

SCFAs mitigate inflammation by enhancing prostacyclin E2 and IL-10 production via epigenetic mechanisms, while also promoting the differentiation of Treg cells and curbing the activity of Th17 cells, thus playing a key role in immune regulation.^47,54^ Zhu et al. found that SCFAs can attenuate mast cell-driven skin inflammation through animal experiments.^27^

Liu et al. used 16s rDNA sequencing and quantitative PCR to identify the flora of patients with CSD. Their comprehensive analysis yielded a reduction in beneficial bacteria producing SCFAs, like *Subdoligranulum* and *Ruminococcus bromii*.^17^ Abnormal cytokine expression and the dysfunction of Treg cells are prevalent in patients with CU.^20^ Th2 cells release cytokines like IL-4, IL-5, and IL-13, which stimulate the production of IgE and can activate mast cells, basophils, and eosinophils. Furthermore, IL-4 enhances the expression of the IgE receptor.^55^ Based on previous evidence, the researchers proposed a hypothesis that the reduction of SCFAs would further suppress the production and function of Treg cells, which leads to an imbalance of the Th1/Th2 ratio and an increase in the production of IL-4 and IgE.^17^ Rezazadeh et al. found that *Lactobacillus* and *Bifidobacterium* may also play a protective role against CU by inhibiting inflammation through the differentiation of Treg cells.^56^

Increased Th17 cells have been detected in patients with CU compared to HCs.^57^ Luo et al. found that the genus *Prevotella* was more abundant in the CSU group than in HCs and was shown to stimulate Th17 immune response and recruit neutrophils to trigger persistent inflammation in mouse experiments.^46^ Zhang et al. observed a notable rise in the relative abundance of the *Proteobacteria* phylum in CSU patients.^19^ This increase in *Proteobacteria* is commonly seen in individuals with asthma and allergic conditions, potentially because *Proteobacteria* can induce allergic responses by upregulating the expression of Th17-related genes.^58^

SCFAs not only contribute to maintain the immune microenvironment but also stabilize the intestinal mucosal epithelial barrier.^59^ Enhancing the diversity of the gut microbiota by lowering pH, maintaining the barrier integrity of the epithelium, and regulating the secretion of mucus may serve as other protective mechanisms.^60,61^ Yüksekal et al. found that the mean fecal pH of CSU patients (7.17) was higher than that of HCs (6.7), which was associated with reduced production of SCFAs.^18^ Lower intestinal pH supports the growth of beneficial bacteria like *Bifidobacterium* and *Lactobacillus*, which in turn helps maintain a healthy pH balance and prevents the colonization of harmful bacteria.^62^

#### 2.2.2. The intensified pro-inflammatory role of LPS

Gram-negative bacteria are the primary constituents of the intestinal flora, and lipopolysaccharides on their cell walls can stimulate IgE-induced MC degranulation and the release of inflammatory mediators through Toll-like receptor 4.^63^ An increase in opportunistic pathogens was discovered in the intestines of CSU patients, while decreased levels of SCFAs led to heightened intestinal mucosal permeability, ultimately resulting in elevated levels of circulating LPS.^27^

An increased abundance of the *Enterobacteriaceae* family, the main opportunistic pathogens producing LPS, was found in the feces of patients with CU, which promotes Th2 cell differentiation and produces IL-4.^20,21^ The discovery was further validated by research conducted by Lu et al., which identified elevated *Escherichia coli*levels within CSU patients.^39^ Zhu et al. also indicated that a high level of *Klebsiella pneumoniae* increased passive cutaneous anaphylactic shock response in recipient mice and raised blood levels of LPS.^27^

#### 2.2.3. The role of parasite infection in CU

Parasitic infections have also been identified as contributors to abnormal intestinal microecology associated with the pathogenesis of CSU. The association between internal parasite infection and CSU has been studied in several researches, summarized by a systematic review.^64^ Patients with CSU were more frequently diagnosed with protozoa and exhibited a significantly elevated risk of *toxocariasis* seropositivity and sensitization to *Anisakis simplex* compared to the HCs. They also showed higher prevalence of *fasciolosis*, *Anisakis simplex* sensitization, and *Blastocystis hominis allele 34* (ST3) compared to controls. Additionally, *Blastocystis hominis* was the most common parasite in children with CSU. After antiparasitic medication, all incorporated children came back to normal.^65^

By analyzing crude extracts and isolated components from whole larvae of Ascarididae (*Anisakis simplex* and *Toxocara canis*), Viñas et al. observed that *Anisakis* appears to play a more significant role than *Toxocara* in the association between urticaria and parasitic infestations, with tropomyosin and Ani s l serving as crucial markers.^66^

Nematodes, a type of helminth, affect millions of individuals worldwide. Various nematode infections have been linked to the development of urticaria and/or angioedema, such as *Dirofilaria spp*, *Enterobius vermicularis*, *Gnathostoma spp* and so on.^67^

Helminth infections may impact the onset and progression of CSU via various pathways involving the activation of mast cells and basophils.^24^ Parasite-specific IgE activates mast cells and basophils via high-affinity IgE receptors (FceRI). Helminths, such as *Toxocara canis* or *Fasciola hepatica*, and protozoa, such as *Blastocystis hominis* or *Giardia lamblia*, have the ability to induce the production of high levels of specific IgE antibodies against their antigens in the host, which can result in sensitization and degranulation of host mast cells.^68,69^

Additionally, helminth parasites disrupt the body’s protective barriers, prompting a Th2 immune response and tissue repair.^64^ And the parasites themselves inhibit the differentiation of Th1 cells and encourage the development of Th2 cells, while B cells aid Th1 responses by producing IgG1–3, forming immune complexes (CIC) with parasite antigens. This process activates the complement system and generates anaphylatoxins C5a and C3a to act on mast cells, contributing to the development of urticaria.^70,71^

#### 2.2.4. Other possible metabolite-related mechanisms

In metabolomics analyses, SCFAs were the predominant metabolites, while unsaturated fatty acids may also be essential in the pathogenesis of CSU. Unsaturated fatty acids such as arachidonic acid and docosahexaenoic acid have been shown to stimulate the growth of *Bacteroides* and have anti-inflammatory effects,^72^ supported by Wang et al. and Luo et al.^26,46^ *Lachnospira* showed a negative correlation with arachidonic acid, while *Gemmiger* exhibited a negative correlation with (±) 8-HETE, a product of the enzymatic oxidation of arachidonic acid.^46,73^ Furthermore, purine metabolites such as adenine and adenosine were also increased in the CSU group. A mouse experiment found that inflammatory cells in allergic mice may result in increased levels of adenine and adenosine, providing further evidence that inflammation contributes to the pathogenesis of CSU.^74^

### 2.3. The interaction between gut microbiome and current treatment

Currently, standardized protocols have been utilized to treat CSU. However, the effectiveness of these treatments for CSU patients can be affected by several factors, including gut flora. Some patients exhibit nsAH resistance, characterized by an inadequate response to any dose of nsAH, posing a significant challenge in CSU management. Patients with nsAH resistance have a more intense intestinal or systemic inflammatory response. It has been speculated that dysbiosis of the intestinal flora may be a contributing factor for nsAH resistance.^75^ Song et al. conducted a study comparing the intestinal flora of CSU patients with and without nsAH resistance. The study found that CSU patients with nsAH resistance had higher levels of genus *Prevotella*, *Megamonas*, *Escherichia*, *Succinivibrio*, *Klebsiella*, *and Colidextribacter*. Conversely, CSU patients without nsAH resistance had lower levels of genus *Blautia*, *Alistipes*, and *Anaerostipes*.^21^ In another study by Liu et al. genus *Lachnospira* was identified as a biomarker for nsAH characteristics.^76^ However, further research is needed to investigate the exact mechanisms.

One meta-analysis revealed that HP may be linked to the development and persistence of CSU.^42^ The efficacy of HP eradication therapy in alleviating CSU symptoms was found to be significant. Interestingly, CSU patients who received antibiotic treatment for HP eradication exhibited a notably higher rate of CSU remission, regardless of HP eradication status. As regard to parasite, majorities of CSU patients with PI were deemed suitable for the antiparasitic therapy.^64^

Furthermore, the current treatment has also been demonstrated to contribute to the alteration of gut microbiome in CSU patients. Following omalizumab therapy, a notable decrease was observed in the relative abundance of *Alphaproteobacteria* and *Betaproteobacteria* at the class level, as well as *Burkholderia*, *Rhodococcus*, and *Sphingomonas* at the genus level.^77^ This reduction may be a contributing factor to the favorable recovery outcomes.

## 3. Approaches targeting gut microbiome for CU therapy

**Figure 3.**
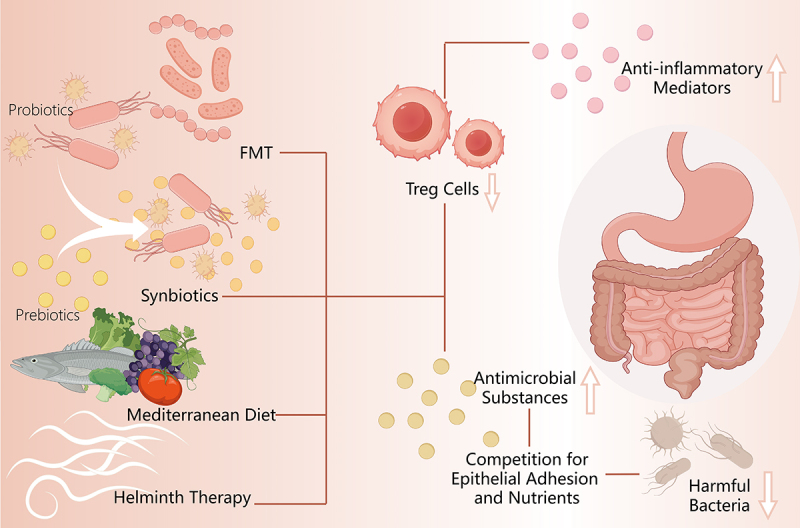
Summary of relevant therapies of chronic urticaria targeted at gut microbiome.

CU poses a common and clinically challenging disorder, with approved therapies effectively managing symptoms but lacking efficacy in altering the natural disease progression.^78^ Therefore, there exists a need for enhanced therapy methodologies, and the correlation between gut microbiota and CU presents a propitious therapeutic pathway. Treating CU by regulating intestinal microecology is currently a prominent research focus. (Figure 3).

Therapeutic approaches targeting gut microbiome in chronic urticaria. Gut microbiome-based therapeutics for CU including probiotics, prebiotics, synbiotics, fecal microbiota transplantation (FMT), and others (helminth therapy and Mediterranean Diet). Treg, regulatory T cells (By Figdraw).

### 3.1. Probiotics, prebiotics, and synbiotics

#### 3.1.1. Probiotics

As defined by the World Health Organization and United Nations Food and Agriculture Organization, probiotics are live microorganisms that are considered safe and provide beneficial effects to the host when administered in sufficient quantities through various immunological, epithelial, and microbiological mechanisms.^79–81^ Several mechanisms can be postulated to elucidate the protective properties of probiotics against chronic urticaria. Firstly, evidence suggests a reduction in both quantity^82^ and functionality^83^ of Treg cells in patients with CU. Nevertheless, several studies indicated that these bacteria and associated compounds could induce the generation of Treg cells to react to dietary antigens.^84–87^ By stimulating the synthesis of anti-inflammatory mediators, this induction reduces inflammation and promotes an anti-inflammatory environment that serves as a protective mechanism against CU.^20^ Moreover, through the secretion of antimicrobial substances, suppression of bacterial toxin production, and competition with pathogens for epithelial adhesion and nutrients, probiotics maintain a well-balanced intestinal bacterial ecology and improve intestinal barrier function, which in turn reduces the production of deleterious metabolites in the body.^88^ Thus, dysregulation may become systemic as a result of alterations in the gut microbiota of patients with CU. This highlights the rationale for contemplating altering of the gut microbiome through the administration of probiotics.

At present, there is substantial and promising evidence concerning the benefits of managing gut microbiota and the use of probiotics across various medical fields, including dermatology.^89–95^ Also, significant evidence implies that the gut microbiota may exert a significant influence on CU. Nonetheless, there remains a scarcity of dedicated case series or trials addressing this specific aspect.

As mentioned above, evidence from Nabizadeh’s and Rezazadeh’s studies suggests that some gut microbiota, indicating a safeguarding influence against CU, have the potential to participate as probiotics in the treatment and mitigation of CU.

Nettis et al. conducted a study to investigate the impact of a combination of two probiotics (*Lactobacillus salivarius LS01* and *Bifidobacterium breve BR03*) on clinical progression in patients with refractory CSU. Assessment of patient improvement following probiotic administration was conducted using the 5-question urticaria quality of life questionnaire and the urticaria activity score over 7 days (UAS7) at baseline and week 8. During the probiotic intervention period, among 38 patients, 9 (23.7%) exhibited mild improvement in clinical symptoms; 1 (2.6%) demonstrated significant improvement; and 1 (2.6%) achieved complete resolution of urticaria symptoms. Symptoms remained unchanged in 27 patients (71.1%). Their findings substantiate the therapeutic efficacy of this probiotic combination in reducing symptomatology scores for certain CSU patients, primarily those with allergic rhinitis symptoms persisting despite treatment with H1 antihistamines, thereby enhancing their overall quality of life without any side effects.^96^

Desloratadine dry suspension was administered to both groups of children in a randomized placebo-controlled study by Bi et al., which involved 206 children with CU. In the treatment group, 104 children were additionally administered Yimingjia® (a lyophilized mixture of six organisms at a concentration of 5 × 10^9^ CFU live total bacteria per gram. After 4 weeks of monitoring, the treatment group exhibited a remarkable reduction in both wheal size and attack frequency, with 80.8% achieving a significant overall response rate (comprising significant improvement and complete response) compared to the 62.5% observed in the placebo group.^97^ This work further confirms the possible effectiveness of particular probiotic strain combinations in addition to provide evidence-based support for the use of probiotics in the treatment of CU.

#### 3.1.2. Prebiotics

Prebiotics refer to supplements or foods containing non-digestible components that not only selectively promote the growth and/or activity of beneficial indigenous probiotic bacteria but also enforce the immune system.^98^

In a prospective, double-blind, placebo-controlled study by the Arslanoglu team, healthy full-term infants at atopic risk were fed hypoallergenic formula with prebiotics (0.8 g/100 ml galactooligosaccharides (scGOS)/fructooligosaccharides (lcFOS)) in 42 and placebo (0.8 g/100 ml maltodextrin) in 50 during the first 6 months of life and subsequently completed a 5-year follow-up. The incidence of allergic urticaria was lower in children in the scGOS/lcFOS group (6%) compared to the placebo group (38%). The protective effect of oligosaccharide prebiotics (scGOS/lcFOS) against allergic urticaria in high-risk infants starting early in life and continuing from infancy to 5 years of age was demonstrated.^99^ In another prospective, randomized, double-blind, placebo-controlled trial conducted by van der’s team, healthy full-term infants of parents with a history of atopic dermatitis were fed hypoallergenic formula and divided into two groups, with prebiotics added to the intervention group (8 g/liter of scGOS/lcFOS), and placebo added to the placebo group (8 g/liter of maltodextrin). At the end of the 6-month intervention period, blinded follow-up continued until the infant was 2 years old. The cumulative incidence of allergic urticaria was higher in the placebo group (10.3%) than in the intervention group (1.5%).^94^ In conclusion, studies related to the application of prebiotics singly for the prevention of urticaria, mainly conducted in infants with scGOS/lcFOS, have demonstrated an effective protective effect. In addition, long-term follow-up studies with a larger population and a wider age range and using a wider variety of prebiotics are necessary to assess the potential preventive effect of such prebiotics on urticaria.

#### 3.1.3. Synbiotics

Combining probiotics and prebiotics that work together synergistically, synbiotics possess properties of both probiotics and prebiotics and are developed to address potential challenges in probiotic survival within the gastrointestinal tract, thereby benefiting the host’s health.^89,100^

Atefi’ s team carried out a blinded randomized controlled clinical trial on 42 patients with CU for 8 weeks. Patients were assigned to control (only antihistamine) and intervention groups (antihistamine + synbiotic) through computerized randomization with a 1:1 allocation ratio. Intervention groups received twice-daily oral probiotic capsules named LactoCare, which contains high amounts of beneficial bacteria including *Lactobacillus rhamnosus*, *Lactobacillus casei*, *Lactobacillus acidophilus*, *Bifidobacterium breve*, *Lactobacillus bulgaricus*, *Bifidobacterium longum*, S*treptococcus thermophilus* and fructooligosaccharides as prebiotic. There was 66% score reduction in the intervention group compared to 53% reduction in the control group. In the control and intervention group improvement of dermatology life quality index was 44% and 66%, respectively. While there wasn’t a significant difference in effectiveness between using probiotics and antihistamines together versus using antihistamines alone based on UAS7 scores, patients receiving the combination therapy may experience a higher reduction in itching, number of hives, and total UAS7 score.^101^

The aforementioned studies reaffirm the efficacy, safety, and satisfaction of probiotics, prebiotics, and synthetic options as adjunctive therapies for managing CSU. These interventions offer effective relief from symptoms and enhance quality of life, exhibiting no apparent adverse effects. Nevertheless, loss to follow-up and short follow-up times are among the major limitations of these studies. Moreover, given the small number of relevant clinical trials and the small sample size included, more randomized controlled trials with blinding of patients are needed. Moreover, comprehensive, and intuitive mechanistic studies regarding the efficacy of probiotics and prebiotics in CU are currently lacking. In addition, the high cost of symbiotic organisms may be a limiting factor for further research on such topics.

### 3.2. Fecal microbiota transplantation

Remarkably effective in altering the gut microbiota, fecal microbiota transplantation (FMT) presents a viable therapeutic approach. The clinical reactions observed in a range of disorders following FMT demonstrate the intricate link between microbiota and host. These problems include, but are not limited to, *Clostridium difficile* infection, inflammatory bowel disease, diabetes mellitus, cancer, liver cirrhosis, and gut-brain disorders.^102^

According to earlier research, FMT has potential for use in dermatological applications. FMT suppresses many representative inflammatory cytokines and has demonstrated a quicker tendency in reversing the skin’s epidermal layer’s increased thickness.^103^ Another study by Kim’s group used mice as experiment subjects and showed that FMT may raise levels of essential SCFAs in gut physiology and restore intestinal microbiota to a donor-like condition. Moreover, FMT decreases IgE levels, balances Th1/Th2 responses, controls gut microbiota-mediated Treg cells, and diminishes mast cells, eosinophils, and basophils, all of which help to lessen allergic reactions in atopic dermatitis.^104^

Wu et al. reported a case in which a female patient who had experienced recurring paroxysmal stomach discomfort with urticaria for six years underwent four sessions of colonic FMT administered by transendoscopic enteral tube. At the one-year follow-up, there was a noticeable improvement in the patient’s symptoms, which allowed the treatment to end and the patient to eventually resume their regular social and eating activities. Furthermore, post-FMT gut microbiota composition underwent considerable alterations, as revealed by 16S rRNA sequencing analysis of fecal samples. These changes included an increase in *Prevotella* abundance and a decrease in *Bacteroides* and *Faecalibacterium* abundance. Additionally, gate-level analysis showed a lower steroidogenic/bacteroidogenic ratio, indicating a change in the gut microbiota of individuals with cystic urealitis and perhaps supporting the maintenance of gut microbiota homeostasis.^105^

In conclusion, the utilization of the microbiota represents the primary paradigm shift in the application of microbial cells. We need more clinical investigations of FMT for CU because the available clinical evidence is currently limited. For the benefit of more CU sufferers, thought needs to be made to standardizing FMT into a practical and dependable therapy option in the meantime.

### 3.3. Other therapeutic strategies

The scientific literature highlights diet as a pivotal factor in molding the gut microbiota, host metabolites, and barrier immune system, contributing to approximately 20% of the variation observed in the human gut microbiome.^106,107^ Changes in diet quickly impact the diversity of the gut microbiota, favoring the proliferation of specific bacterial populations while influencing gut pH, intestinal permeability, and the production of bacterial metabolites, potentially triggering inflammation.^108^ However, stressing that food quality and nutrients – rather than quantity – are thought to be the main determinants of a good or unhealthy diet is important.^109^ Currently, studies have shown that certain foods can alter the composition of gut microbiota and alleviate symptoms of dermatologic symptoms, like olive-derived antioxidant dietary fiber.^110^

There are studies suggesting that the Mediterranean diet may influence the gut microbiota, which in turn affects the CU. The Mediterranean diet, characterized by its healthful eating habits, has been linked to decreased chances of developing cardiovascular diseases and metabolic syndrome.^111^ This eating regimen is marked by a blend of intricate carbohydrates rich in fiber, polyunsaturated fatty acids known for their antiatherogenic and anti-inflammatory effects, and bioactive compounds boasting antioxidant properties like flavonoids, phytosterols, terpenes, and polyphenols, serving as an exemplar of nutritious eating.^112^ A study revealed that the mean Mediterranean diet score was 5.40 ± 1.88 in the group of patients with CSU, compared to 6.30 ± 1.39 in the HCs (*p* < 0.001). The urticaria activity score in CSU patients over 7 days was inversely associated with the Mediterranean diet score, while the urticaria control test score was positively correlated.^113^ The study suggests that the Mediterranean diet’s anti-inflammatory and antioxidant properties may help alleviate systemic inflammation and oxidative stress,^114^ which contribute to the pathogenesis of CSU disease. In addition, adherence to the Mediterranean diet is linked to the restoration of balanced gut microbiota, with increases observed in *Bacteroidetes* and specific beneficial *Clostridium* groups, while decreases are noted in *Proteobacteria* and *Bacillaceae* phyla.^115^ Western diets drive an imbalanced gut microbiota, marked by an increased *Firmicutes*: *Bacteroidetes* ratio. Conversely, the Mediterranean diet fosters the growth of beneficial bacteria and their metabolites, while mitigating dysbiosis and reducing levels of LPS.^116^ MD pattern was also characterized by greater diversity, better intestinal barrier function and permeability.^117^

Despite the fact that it may play a crucial part in the composition and activity of the gut microbiota and the ensuing immunological and inflammatory responses, there is surprisingly little data about how dietary diversity affects the microbiome of patients with convalescent units. To better understand the role of dietary, environmental, and lifestyle factors that may lead to immunological dysfunction, CU etiology must be thoroughly explored. There is a need to bring together the dietary diversity and dermatologic expertise of dietitians, nutritionists, immunologists, microbiologists, dermatopathologists, and biotechnologists in well-defined methodologies for well-conducted randomized controlled trials.

In addition, some adjuvant therapies further influence the course of the disease by affecting the species and quantity of gut bacteria, thus providing insights into CU symptom remission. Helminth therapy, as an allergy immunotherapy, has been found to be therapeutic for a selection of immune and inflammatory diseases like rheumatoid arthritis^118^ and multiple sclerosis.^119^ Strong evidence from animal studies demonstrates that worms not only reduce parasite-specific immune responses but also modulate their own immunity and allergic inflammatory responses, improving metabolic balance.^120^ Active infection with *Ascaris lumbricoides* appears to confer protection against atopic dermatitis in Cuban children, whereas previous infection with *Enterobius vermicularis*.^121^

The word “helminth” comes from the Greek word “helmins,” meaning “worm.” Species categorized as *annelids* (segmented worms), *nematodes* (roundworms), *trematodes* (flukes), and *cestodes* (tapeworms) are the four primary families of human helminths.^122^ The two ways in which helminth therapy works are (1) directly through immune system influence, and (2) by alteration of the gut microbiota,^123^ which has an effect on the organism. Helminths evade immunological recognition and confrontation by manipulating the animals’ natural immune responses. Pre-existing allergy or immunological-related conditions in the host may go into remission or potentially go away completely as a result of this immune adjustment.^124^ In another research, Paneth cells and Goblet cells produce small proline-rich protein 2A after helminth infection, which is triggered by type 2 immunity.^125^

However, further evidence in larger studies is needed to prove or disprove their safety and efficacy. Probiotics, prebiotics, synbiotics, nutrition, and FMT that target the gut microbiota have been demonstrated in many organic disease models and/or clinical studies to have preventative potential. More focus and acceptance should be placed on methods for using microbiota in clinical and scientific research. Even while research on other treatment approaches, such as helminth therapy, which targets the gut microbiota, is continuing and shows promise, its processes are yet unknown. Clinical trials often have limited sample sizes, which makes it difficult to evaluate safety and efficacy impartially. Moreover, barriers to the clinical use of linked drugs and production costs continue to exist. These analog medications are difficult to make, which indicates that they won’t be easily accessible for clinical usage very soon.

## 4. Conclusion

The composition and alterations of the gut microbiome have a significant impact on the pathological state of CU, and analysis of these host-bacteria interactions could enhance our comprehension of the mechanisms through which they impact and influence the progression of CU. By identifying these mechanisms and targeting the intestinal microbiome, researchers hope to develop new therapeutic strategies such as probiotics, prebiotics, synbiotics and FMT. We believe that these gut bacteria-based therapeutic strategies have great future potential for application in the treatment and prevention of CU. Researches on the exact mechanism of the gut microbiota in CU is still insufficient and it is hoped that researchers will in the future clarify the exact mechanism of the gut microbiota in the pathogenesis of CU as a basis for high quality clinical research, especially applied research.

## Abbreviations

CU: chronic urticaria
nsAH: non-sedating H1-antihistamines
TNF: tumor necrosis factor
IL: interleukin
Th: T helper cells
CSU: chronic spontaneous urticaria
HCs: healthy controls
PI: parasite infection
PCR: polymerase chain reaction
HP: Helicobacter pylori
SCFAs: short-chain fatty acids
CSD: Chronic Spontaneous Urticaria and Symptomatic Dermographism
Treg cells: regulatory T cells
LPS: Lipopolysaccharide
CIC: immune complexes
scHOS: galactooligosaccharides
lcFOS: fructooligosaccharides
FMT: fecal microbiota transplantation

## Data Availability

This study did not generate new data but was a review of the literature.

## References

[1] Zuberbier T, Abdul Latiff AH, Abuzakouk M, Aquilina S, Asero R, Baker D, Ballmer‐Weber B, Bangert C, Ben‐Shoshan M, Bernstein JA, et al. The international EAACI/GA²LEN/EuroGuiDerm/APAAACI guideline for the definition, classification, diagnosis, and management of urticaria. Allergy. 2022;77(3):734–20. doi:10.1111/all.15090. 34536239

[2] Lang DM, Ropper AH. Chronic urticaria. N Engl J Med. 2022;387(9):824–831. doi:10.1056/NEJMra2120166. 36053507

[3] Gonçalo M, Gimenéz‐Arnau A, Al‐Ahmad M, Ben‐Shoshan M, Bernstein JA, Ensina LF, Fomina D, Galvàn CA, Godse K, Grattan C, et al. The global burden of chronic urticaria for the patient and society*. Br J Dermatol. 2021;184(2):226–236. doi:10.1111/bjd.19561. 32956489

[4] Zhou B, Li J, Liu R, Zhu L, Peng C. The role of crosstalk of immune cells in pathogenesis of chronic spontaneous urticaria. Front Immunol. 2022;13:879754. doi:10.3389/fimmu.2022.879754. 35711438 PMC9193815

[5] Giménez-Arnau AM, DeMontojoye L, Asero R, Cugno M, Kulthanan K, Yanase Y, Hide M, Kaplan AP. The pathogenesis of chronic spontaneous urticaria: the role of infiltrating cells. J Allergy Clin Immunol Pract. 2021;9(6):2195–2208. doi:10.1016/j.jaip.2021.03.033. 33823316

[6] Elieh-Ali-Komi D, Metz M, Kolkhir P, Kocatürk E, Scheffel J, Frischbutter S, Terhorst-Molawi D, Fox L, Maurer M. Chronic urticaria and the pathogenic role of mast cells. Allergol Int. 2023;72(3):359–368. doi:10.1016/j.alit.2023.05.003. 37210251

[7] Altrichter S, Frischbutter S, Fok JS, Kolkhir P, Jiao Q, Skov PS, Metz M, Church MK, Maurer M. The role of eosinophils in chronic spontaneous urticaria. J Allergy Clin Immunol. 2020;145(6):1510–1516. doi:10.1016/j.jaci.2020.03.005. 32224275

[8] Saini SS. Urticaria and basophils. Allergol Int. 2023;72(3):369–374. doi:10.1016/j.alit.2023.05.001. 37221123

[9] Kulthanan K, Tuchinda P, Chularojanamontri L, Chanyachailert P, Korkij W, Chunharas A, Wananukul S, Limpongsanurak W, Benjaponpitak S, Wisuthsarewong W, et al. Clinical practice guideline for diagnosis and management of urticaria. Asian Pac J Allergy Immunol. 34:190–200. 27690471

[10] Criado PR, Maruta CW, Alchorne ADODA, Ramos AMC, Gontijo B, Santos JBD, Martins LEAM, Rivitti-Machado MC, Silvares MRC, Pires MC, et al. Consensus on the diagnostic and therapeutic management of chronic spontaneous urticaria in adults - Brazilian society of dermatology. An Bras Dermatol. 2019;94(2 suppl 1):56–66. doi:10.1590/abd1806-4841.2019940209. PMC6544033 31166404

[11] Kulthanan K, Chaweekulrat P, Komoltri C, Hunnangkul S, Tuchinda P, Chularojanamontri L, Maurer M. Cyclosporine for chronic spontaneous urticaria: a meta-analysis and systematic review. J Allergy Clin Immunol Pract. 2018;6(2):586–599. doi:10.1016/j.jaip.2017.07.017. 28916431

[12] Casale TB. Novel biologics for treatment of chronic spontaneous urticaria. J Allergy Clin Immunol. 2022;150(6):1256–1259. doi:10.1016/j.jaci.2022.06.027. 36180286

[13] Giménez-Arnau AM, Salman A. Targeted therapy for chronıc spontaneous urtıcarıa: ratıonale and recent progress. Drugs. 2020;80(16):1617–1634. doi:10.1007/s40265-020-01387-9. 32857360

[14] Mahmud M, Akter S, Tamanna SK, Mazumder L, Esti IZ, Banerjee S, Akter S, Hasan M, Acharjee M, Hossain M, et al. Impact of gut microbiome on skin health: gut-skin axis observed through the lenses of therapeutics and skin diseases. Gut Microbes. 2022;14(1):2096995. doi:10.1080/19490976.2022.2096995. 35866234 PMC9311318

[15] Routy B, Jackson T, Mählmann L, Baumgartner CK, Blaser M, Byrd A, Corvaia N, Couts K, Davar D, Derosa L, et al. Melanoma and microbiota: Current understanding and future directions. Cancer Cell. 2024;42(1):16–34. doi:10.1016/j.ccell.2023.12.003. 38157864 PMC11096984

[16] Su Y-J, Luo S-D, Hsu C-Y, Kuo H-C. Differences in gut microbiota between allergic rhinitis, atopic dermatitis, and skin urticaria: a pilot study. Med (Baltim). 2021;100(9):e25091. doi:10.1097/MD.0000000000025091. PMC7939153 33655988

[17] Liu R, Peng C, Jing D, Xiao Y, Zhu W, Zhao S, Zhang J, Chen X, Li J. Biomarkers of gut microbiota in chronic spontaneous urticaria and symptomatic dermographism. Front Cell Infect Microbiol. 2021;11:703126. doi:10.3389/fcimb.2021.703126. 34858864 PMC8630658

[18] Yuksekal G, Sevimli Dikicier B, Koku Aydin B, Yilmaz K, Altindis M, Koroglu M. Investigation of intestinal microbiome in chronic spontaneous urticaria patients. Int J Dermatol. 2022;61(8):988–994. doi:10.1111/ijd.16054. 35100439

[19] Zhang X, Zhang J, Chu Z, Shi L, Geng S, Guo K. Gut microbiome alterations and functional prediction in chronic spontaneous urticaria patients. J Microbiol Biotechnol. 2021;31(5):747–755. doi:10.4014/jmb.2012.12022. 33746191 PMC9723274

[20] Nabizadeh E, Jazani NH, Bagheri M, Shahabi S. Association of altered gut microbiota composition with chronic urticaria. Ann Allergy Asthma Immunol. 2017;119(1):48–53. doi:10.1016/j.anai.2017.05.006. 28668239

[21] Song Y, Dan K, Yao Z, Yang X, Chen B, Hao F. Altered gut microbiota in H1-antihistamine-resistant chronic spontaneous urticaria associates with systemic inflammation. Front Cell Infect Microbiol. 2022;12:831489. doi:10.3389/fcimb.2022.831489. 35372130 PMC8967245

[22] Kolkhir P, Church MK, Weller K, Metz M, Schmetzer O, Maurer M. Autoimmune chronic spontaneous urticaria: what we know and what we do not know. J Allergy Clin Immunol [Internet]. 2017;139(6):1772–1781.e1. https://pubmed.ncbi.nlm.nih.gov/27777182. 27777182 10.1016/j.jaci.2016.08.050

[23] Brodell LA, Beck LA, Saini SS. Pathophysiology of chronic urticaria. Ann Allergy Asthma Immunol Off Publ Am Coll Allergy Asthma Immunol. 2008;100(4):291–298. doi:10.1016/S1081-1206(10)60588-1. 18450112

[24] Kolkhir P, Giménez-Arnau AM, Kulthanan K, Peter J, Metz M, Maurer M. Urticaria. Nat Rev Dis Primer. 2022;8(1):61. doi:10.1038/s41572-022-00389-z. 36109590

[25] Salem I, Ramser A, Isham N, Ghannoum MA. The gut microbiome as a major regulator of the gut-skin axis. Front Microbiol. 2018;9:1459. doi:10.3389/fmicb.2018.01459. 30042740 PMC6048199

[26] Wang D, Guo S, He H, Gong L, Cui H. Gut microbiome and serum metabolome analyses identify unsaturated fatty acids and butanoate metabolism induced by gut microbiota in patients with chronic spontaneous urticaria. Front Cell Infect Microbiol. 2020;10:24. doi:10.3389/fcimb.2020.00024. 32154184 PMC7047433

[27] Zhu L, Jian X, Zhou B, Liu R, Muñoz M, Sun W, Xie L, Chen X, Peng C, Maurer M, et al. Gut microbiota facilitate chronic spontaneous urticaria. Nat Commun. 2024;15(1):112. doi:10.1038/s41467-023-44373-x. 38168034 PMC10762022

[28] Loo EXL, Chew LJM, Zulkifli AB, Ta LDH, Kuo IC, Goh A, Teoh OH, Van Bever H, Gluckman PD, Yap F, et al. Comparison of microbiota and allergen profile in house dust from homes of allergic and non-allergic subjects- results from the GUSTO study. World Allergy Organ J. 2018;11:37. doi:10.1186/s40413-018-0212-5. 30534340 PMC6280478

[29] Wang X, Yi W, He L, Luo S, Wang J, Jiang L, Long H, Zhao M, Lu Q. Abnormalities in gut microbiota and metabolism in patients with chronic spontaneous urticaria. Front Immunol. 2021;12:691304. doi:10.3389/fimmu.2021.691304. 34721374 PMC8554312

[30] Kern L, Abdeen SK, Kolodziejczyk AA, Elinav E. Commensal inter-bacterial interactions shaping the microbiota. Curr Opin Microbiol. 2021;63:158–171. doi:10.1016/j.mib.2021.07.011. 34365152

[31] Leung JM, Graham AL, Knowles SCL. Parasite-microbiota interactions with the vertebrate gut: synthesis through an ecological lens. Front Microbiol. 2018;9:843. doi:10.3389/fmicb.2018.00843. 29867790 PMC5960673

[32] Zuberbier T, Aberer W, Asero R, Bindslev-Jensen C, Brzoza Z, Canonica GW, Church MK, Ensina LF, Giménez-Arnau A, Godse K, et al. The EAACI / GA 2 LEN / EDF / WAO guideline for the definition, classification, diagnosis, and management of urticaria: the 2013 revision and update. Allergy. 2014;69(7):868–887. doi:10.1111/all.12313. 24785199

[33] Brar NK, Ramly B, O’Connor C, Lucey J. A case report on norovirus related urticarial rash in paediatric patient. Arch Dis Child. 2019;104:A56.

[34] Yasuda M, Shoji K, Tomita K, Uchida Y, Uematsu S, Yoshida K, Kono N, Funatsu M, Miyairi I. Clinical and laboratory diagnosis of exanthems among Japanese children younger than 6 years old in the post–measles-Rubella vaccine era. Pediatr Infect Dis J. 2024;43(2):e44–8. doi:10.1097/INF.0000000000004175. 37963264

[35] Bilbao A, Garcia JM, Pocheville I, Gutierrez C, Corral JM, Samper A, Rubio G, Benito J, Villas P, Fernandez D, et al. [Round Table: Urticaria in relation to infections]. Allergol Immunopathol (Madr). 1999;27(2):73–85. 10354011

[36] Staubach P, Vonend A, Burow G, Metz M, Magerl M, Maurer M. Patients with chronic urticaria exhibit increased rates of sensitisation to Candida albicans, but not to common moulds. Mycoses. 2009;52(4):334–338. doi:10.1111/j.1439-0507.2008.01601.x. 18793264

[37] James J, Warin RP. An assessment of the role of Candida albicans and food yeasts in chronic urticaria. Br J Dermatol. 1971;84(3):227–237. doi:10.1111/j.1365-2133.1971.tb14212.x. 5572675

[38] Candela M, Rampelli S, Turroni S, Severgnini M, Consolandi C, De Bellis G, Masetti R, Ricci G, Pession A, Brigidi P. Unbalance of intestinal microbiota in atopic children. BMC Microbiol. 2012;12(1):95. doi:10.1186/1471-2180-12-95. 22672413 PMC3404014

[39] Lu T, Chen Y, Guo Y, Sun J, Shen W, Yuan M, Zhang S, He P, Jiao X. Altered gut microbiota diversity and composition in chronic urticaria. Markers. 2019;2019:6417471. doi:10.1155/2019/6417471. PMC6881578 31827639

[40] Shi YZ, Tao QF, Qin HY, Li Y, Zheng H. Causal relationship between gut microbiota and urticaria: a bidirectional two-sample mendelian randomization study. Front Microbiol. 2023;14:1189484. doi:10.3389/fmicb.2023.1189484. 37426010 PMC10324650

[41] Gu H, Li L, Gu M, Zhang G. Association between Helicobacter pylori Infection and Chronic Urticaria: A Meta-Analysis. Gastroenterol Res Pr. 2015;2015:486974. doi:10.1155/2015/486974. PMC4378606 25861258

[42] Kim HJ, Kim YJ, Lee HJ, Hong JY, Park AY, Chung EH, Lee SY, Lee JS, Park YL, Lee SH, et al. Systematic review and meta-analysis: effect of Helicobacter pylori eradication on chronic spontaneous urticaria. Helicobacter. 2019;24(6):e12661. doi:10.1111/hel.12661. 31523897

[43] Tan RJ, Sun HQ, Zhang W, Yuan HM, Li B, Yan HT, Lan CH, Yang J, Zhao Z, Wu JJ, et al. A 21–35 kDa Mixed Protein Component from Helicobacter pylori Activates Mast Cells Effectively in Chronic Spontaneous Urticaria. Helicobacter. 2016;21(6):565–574. doi:10.1111/hel.12312. 27061753

[44] Tsai CC, Kuo TY, Hong ZW, Yeh YC, Shih KS, Du SY, Fu HW. Helicobacter pylori neutrophil-activating protein induces release of histamine and interleukin-6 through G protein-mediated MAPKs and PI3K/Akt pathways in HMC-1 cells. Virulence. 2015;6(8):755–765. doi:10.1080/21505594.2015.1043505. 26375619 PMC4826132

[45] Zafar H, Saier MH. Gut Bacteroides species in health and disease. Gut Microbes. 2021;13(1):1–20. doi:10.1080/19490976.2020.1848158. PMC7872030 33535896

[46] Luo Z, Jin Z, Tao X, Wang T, Wei P, Zhu C, Wang Z. Combined microbiome and metabolome analysis of gut microbiota and metabolite interactions in chronic spontaneous urticaria. Front Cell Infect Microbiol. 2022;12:1094737. doi:10.3389/fcimb.2022.1094737. 36710970 PMC9874702

[47] Smith PM, Howitt MR, Panikov N, Michaud M, Gallini CA, Bohlooly-Y M, Glickman JN, Garrett WS. The microbial metabolites, short-chain fatty acids, regulate colonic Treg cell homeostasis. Science. 2013;341(6145):569–573. doi:10.1126/science.1241165. 23828891 PMC3807819

[48] Rinninella E, Raoul P, Cintoni M, Franceschi F, Miggiano GAD, Gasbarrini A, Mele MC. What is the Healthy Gut Microbiota Composition? A Changing Ecosystem across Age, Environment, Diet, and Diseases. Microorganisms. 2019;7(1):14. doi:10.3390/microorganisms7010014. 30634578 PMC6351938

[49] Furusawa Y, Obata Y, Fukuda S, Endo TA, Nakato G, Takahashi D, Nakanishi Y, Uetake C, Kato K, Kato T, et al. Commensal microbe-derived butyrate induces the differentiation of colonic regulatory T cells. Nature. 2013;504(7480):446–450. doi:10.1038/nature12721. 24226770

[50] Ćesić D, Lugović Mihić L, Ozretić P, Lojkić I, Buljan M, Šitum M, Zovak M, Vidović D, Mijić A, Galić N, et al. Association of Gut Lachnospiraceae and Chronic Spontaneous Urticaria. Life Basel Switz. 2023;13(6):1280. doi:10.3390/life13061280. PMC10301119 37374063

[51] Shtessel M, Limjunyawong N, Oliver ET, Chichester K, Gao L, Dong X, Saini SS. MRGPRX2 activation causes increased skin reactivity in patients with chronic spontaneous urticaria. J Invest Dermatol. 2021;141(3):678–681.e2. doi:10.1016/j.jid.2020.06.030. 32771471 PMC11658616

[52] Bedard PM, Brunet C, Pelletier G, Hebert J. Increased compound 48/80 induced local histamine release from nonlesional skin of patients with chronic urticaria. J Allergy Clin Immunol. 1986;78(6):1121–1125. doi:10.1016/0091-6749(86)90260-5. 2431027

[53] Halova I, Ronnberg E, Draberova L, Vliagoftis H, Nilsson GP, Draber P. Changing the threshold—Signals and mechanisms of mast cell priming. Immunol Rev. 2018;282(1):73–86. doi:10.1111/imr.12625. 29431203

[54] Folkerts J, Redegeld F, Folkerts G, Blokhuis B, van den Berg MPM, de Bruijn MJW, van I, Junt T, Tam SY, Galli SJ, et al. Butyrate inhibits human mast cell activation via epigenetic regulation of FcεRI-mediated signaling. Allergy. 2020;75(8):1966–1978. doi:10.1111/all.14254. 32112426 PMC7703657

[55] Kolkhir P, Muñoz M, Asero R, Ferrer M, Kocatürk E, Metz M, Xiang Y-K, Maurer M. Autoimmune chronic spontaneous urticaria. J Allergy Clin Immunol. 2022;149(6):1819–1831. doi:10.1016/j.jaci.2022.04.010. 35667749

[56] Rezazadeh A, Shahabi S, Bagheri M, Nabizadeh E, Jazani NH. The protective effect of Lactobacillus and Bifidobacterium as the gut microbiota members against chronic urticaria. Int Immunopharmacol. 2018;59:168–173. doi:10.1016/j.intimp.2018.04.007. 29655058

[57] Moy AP, Murali M, Nazarian RM. Identification of a Th2- and Th17-skewed immune phenotype in chronic urticaria with Th22 reduction dependent on autoimmunity and thyroid disease markers. J Cutan Pathol. 2016;43(4):372–378. doi:10.1111/cup.12673. 26785710

[58] Huang YJ, Nelson CE, Brodie EL, Desantis TZ, Baek MS, Liu J, Woyke T, Allgaier M, Bristow J, Wiener-Kronish JP, et al. Airway microbiota and bronchial hyperresponsiveness in patients with suboptimally controlled asthma. J Allergy Clin Immunol. 2011;127(2):372–381.e3. doi:10.1016/j.jaci.2010.10.048. 21194740 PMC3037020

[59] Liu P, Wang Y, Yang G, Zhang Q, Meng L, Xin Y, Jiang X. The role of short-chain fatty acids in intestinal barrier function, inflammation, oxidative stress, and colonic carcinogenesis. Pharmacol Res. 2021;165:105420. doi:10.1016/j.phrs.2021.105420. 33434620

[60] Laval L, Martin R, Natividad JN, Chain F, Miquel S, Desclee de Maredsous C, Capronnier S, Sokol H, Verdu EF, van Hylckama Vlieg JE, et al. Lactobacillus rhamnosus CNCM I-3690 and the commensal bacterium Faecalibacterium prausnitzii A2-165 exhibit similar protective effects to induced barrier hyper-permeability in mice. Gut Microbes. 2015;6(1):1–9. doi:10.4161/19490976.2014.990784. 25517879 PMC4615674

[61] Moratalla A, Gomez-Hurtado I, Santacruz A, Moya A, Peiro G, Zapater P, Gonzalez-Navajas JM, Gimenez P, Such J, Sanz Y, et al. Protective effect of Bifidobacterium pseudocatenulatum CECT7765 against induced bacterial antigen translocation in experimental cirrhosis. Liver Int. 2014;34(6):850–858. doi:10.1111/liv.12380. 24267920

[62] de Moraes JG, Motta ME, Beltrao MF, Salviano TL, da Silva GA. Fecal Microbiota and Diet of Children with Chronic Constipation. Int J Pediatr. 2016;2016:6787269. doi:10.1155/2016/6787269. 27418934 PMC4935906

[63] Poplutz M, Levikova M, Luscher-Firzlaff J, Lesina M, Algul H, Luscher B, Huber M. Endotoxin tolerance in mast cells, its consequences for IgE-mediated signalling, and the effects of BCL3 deficiency. Sci Rep. 2017;7(1):4534. doi:10.1038/s41598-017-04890-4. 28674400 PMC5495797

[64] Kolkhir P, Balakirski G, Merk HF, Olisova O, Maurer M. Chronic spontaneous urticaria and internal parasites – a systematic review. Allergy. 2016;71(3):308–322. doi:10.1111/all.12818. 26648083

[65] Arik Yilmaz E, Karaatmaca B, Sackesen C, Sahiner UM, Cavkaytar O, Sekerel BE, Soyer O. Parasitic Infections in Children with Chronic Spontaneous Urticaria. Int Arch Allergy Immunol. 2016;171(2):130–135. doi:10.1159/000450953. 27907915

[66] Viñas M, Postigo I, Suñén E, Martínez J, Periago MV. Urticaria and silent parasitism by Ascaridoidea: component-resolved diagnosis reinforces the significance of this association. PLOS Negl Trop Dis. 2020;14(4):e0008177. doi:10.1371/journal.pntd.0008177. 32243436 PMC7170265

[67] Minciullo PL, Cascio A, Gangemi S. Association between urticaria and nematode infections. Allergy Asthma Proc. 2018;39(2):86–95. doi:10.2500/aap.2018.38.4104. 29490766

[68] Demirci M, Yildirim M, Aridogan BC, Baysal V, Korkmaz M. Tissue parasites in patients with chronic urticaria. J Dermatol. 2003;30(11):777–781. doi:10.1111/j.1346-8138.2003.tb00477.x. 14684933

[69] Aykur M, Camyar A, Türk BG, Sin AZ, Dagci H. Evaluation of association with subtypes and alleles of Blastocystis with chronic spontaneous urticaria. Acta Trop. 2022;231:106455. doi:10.1016/j.actatropica.2022.106455. 35413246

[70] Mishra PK, Palma M, Bleich D, Loke P, Gause WC. Systemic impact of intestinal helminth infections. Mucosal Immunol. 2014;7(4):753–762. doi:10.1038/mi.2014.23. 24736234

[71] Wc G, Ta W, Je A. Type 2 immunity and wound healing: evolutionary refinement of adaptive immunity by helminths. Nat Rev Immunol [Internet]. 2013 [cited 2024 Apr 19]. 13(8):607–614. https://pubmed.ncbi.nlm.nih.gov/23827958/. 23827958 10.1038/nri3476 PMC3789590

[72] Mirjafari Tafti ZS, Moshiri A, Ettehad Marvasti F, Tarashi S, Sadati Khalili SF, Motahhary A, Fateh A, Vaziri F, Ahmadi Badi S, Siadat SD. The effect of saturated and unsaturated fatty acids on the production of outer membrane vesicles from Bacteroides fragilis and Bacteroides thetaiotaomicron. Gastroenterol Hepatol Bed Bench. 2019;12(2):155–162. 31191841 PMC6536021

[73] Miles EA, Childs CE, Calder PC. Long-Chain Polyunsaturated Fatty Acids (LCPUFAs) and the developing immune system: a narrative review. Nutrients. 2021;13(1):247. doi:10.3390/nu13010247. 33467123 PMC7830895

[74] Moon HG, Tae YM, Kim YS, Gyu Jeon S, Oh SY, Song Gho Y, Zhu Z, Kim YK. Conversion of Th17-type into Th2-type inflammation by acetyl salicylic acid via the adenosine and uric acid pathway in the lung. Allergy. 2010;65(9):1093–1103. doi:10.1111/j.1398-9995.2010.02352.x. 20337611

[75] Maurer M, Staubach P, Raap U, Richter-Huhn G, Bauer A, Ruëff F, Jakob T, Yazdi AS, Mahler V, Wagner N, et al. H1-antihistamine-refractory chronic spontaneous urticaria: it’s worse than we thought – first results of the multicenter real-life AWARE study. Clin Exp Allergy. 2017;47(5):684–692. doi:10.1111/cea.12900. 28160338

[76] Liu R, Peng C, Jing D, Xiao Y, Zhu W, Zhao S, Zhang J, Chen X, Li J. Lachnospira is a signature of antihistamine efficacy in chronic spontaneous urticaria. Exp Dermatol. 2022;31(2):242–247. doi:10.1111/exd.14460. 34558729

[77] Wang M, Zhao L, Wang K, Qin Y, Jin J, Wang D, Yan H, You C. Changes of Gut Microbiome in Adolescent Patients with Chronic Spontaneous Urticaria After Omalizumab Treatment. Clin Cosmet Investig Dermatol. 2023;16:345–357. doi:10.2147/CCID.S393406. PMC9907007 36762258

[78] Kaplan A, Lebwohl M, Giménez-Arnau AM, Hide M, Armstrong AW, Maurer M. Chronic spontaneous urticaria: Focus on pathophysiology to unlock treatment advances. Allergy. 2023;78(2):389–401. doi:10.1111/all.15603. 36448493

[79] Hill C, Guarner F, Reid G, Gibson GR, Merenstein DJ, Pot B, Morelli L, Canani RB, Flint HJ, Salminen S, et al. Expert consensus document. The International Scientific Association for Probiotics and Prebiotics consensus statement on the scope and appropriate use of the term probiotic. Nat Rev Gastroenterol Hepatol. 2014;11(8):506–514. doi:10.1038/nrgastro.2014.66. 24912386

[80] Indian Council of Medical Research Task Force, Co-ordinating Unit ICMR, Co-ordinating Unit DBT. ICMR-DBT guidelines for evaluation of probiotics in food. Indian J Med Res. 2011;134(1):22–25. 21808130 PMC3171912

[81] Oelschlaeger TA. Mechanisms of probiotic actions – A review. Int J Med Microbiol IJMM. 2010;300(1):57–62. doi:10.1016/j.ijmm.2009.08.005. 19783474

[82] Arshi S, Babaie D, Nabavi M, Tebianian M, Ghalehbaghi B, Jalali F, Ahmadvand A, Gholami R. Circulating level of CD4+ CD25+ FOXP3+ T cells in patients with chronic urticaria. Int J Dermatol. 2014;53(12):e561–566. doi:10.1111/ijd.12630. 25311400

[83] Chen WC, Chiang BL, Liu HE, Leu SJ, Lee YL. Defective functions of circulating CD4+CD25+ and CD4+CD25− T cells in patients with chronic ordinary urticaria. J Dermatol Sci. 2008;51(2):121–130. doi:10.1016/j.jdermsci.2008.02.012. 18440785

[84] Chiba T, Seno H. Indigenous clostridium species regulate systemic immune responses by induction of colonic regulatory T cells. Gastroenterology. 2011;141(3):1114–1116. doi:10.1053/j.gastro.2011.07.013. 21794835

[85] Atarashi K, Tanoue T, Shima T, Imaoka A, Kuwahara T, Momose Y, Cheng G, Yamasaki S, Saito T, Ohba Y, et al. Induction of colonic regulatory T cells by indigenous Clostridium species. Science. 2011;331(6015):337–341. doi:10.1126/science.1198469. 21205640 PMC3969237

[86] Nagano Y, Itoh K, Honda K. The induction of Treg cells by gut-indigenous Clostridium. Curr Opin Immunol. 2012;24(4):392–397. doi:10.1016/j.coi.2012.05.007. 22673877

[87] Atarashi K, Tanoue T, Oshima K, Suda W, Nagano Y, Nishikawa H, Fukuda S, Saito T, Narushima S, Hase K, et al. Treg induction by a rationally selected mixture of Clostridia strains from the human microbiota. Nature. 2013;500(7461):232–236. doi:10.1038/nature12331. 23842501

[88] Markowiak P, Śliżewska K. Effects of Probiotics, Prebiotics, and Synbiotics on Human Health. Nutrients. 2017;9(9):1021. doi:10.3390/nu9091021. 28914794 PMC5622781

[89] Aldaghi M, Tehrani H, Karrabi M, Abadi FS, Sahebkar M. The effect of multistrain synbiotic and vitamin D3 supplements on the severity of atopic dermatitis among infants under 1 year of age: a double-blind, randomized clinical trial study. J Dermatol Treat. 2022;33(2):812–817. doi:10.1080/09546634.2020.1782319. 32530339

[90] Dissanayake E, Tani Y, Nagai K, Sahara M, Mitsuishi C, Togawa Y, Suzuki Y, Nakano T, Yamaide F, Ohno H, et al. Skin Care and Synbiotics for Prevention of Atopic Dermatitis or Food Allergy in Newborn Infants: A 2 × 2 Factorial, Randomized, Non-Treatment Controlled Trial. Int Arch Allergy Immunol. 2019;180(3):202–211. doi:10.1159/000501636. 31394530

[91] Passeron T, Lacour J-P, Fontas E, Ortonne J-P. Prebiotics and synbiotics: two promising approaches for the treatment of atopic dermatitis in children above 2 years. Allergy. 2006;61(4):431–437. doi:10.1111/j.1398-9995.2005.00956.x. 16512804

[92] Anania C, Brindisi G, Martinelli I, Bonucci E, D’Orsi M, Ialongo S, Nyffenegger A, Raso T, Spatuzzo M, De Castro G, et al. Probiotics Function in Preventing Atopic Dermatitis in Children. Int J Mol Sci. 2022;23(10):23. doi:10.3390/ijms23105409. PMC9141149 35628229

[93] Fang Z, Li L, Zhang H, Zhao J, Lu W, Chen W. Gut Microbiota, Probiotics, and Their Interactions in Prevention and Treatment of Atopic Dermatitis: A Review. Front Immunol. 2021;12:720393. doi:10.3389/fimmu.2021.720393. 34335634 PMC8317022

[94] van der Aa LB, Heymans HS, van Aalderen WM, Sillevis Smitt JH, Knol J, Ben Amor K, Goossens DA, Sprikkelman AB. Effect of a new synbiotic mixture on atopic dermatitis in infants: a randomized-controlled trial. Clin Exp Allergy J Br Soc Allergy Clin Immunol. 2010;40(5):795–804. doi:10.1111/j.1365-2222.2010.03465.x. 20184604

[95] Gerasimov SV, Vasjuta VV, Myhovych OO, Bondarchuk LI. Probiotic supplement reduces atopic dermatitis in preschool children: a randomized, double-blind, placebo-controlled, clinical trial. Am J Clin Dermatol. 2010;11(5):351–361. doi:10.2165/11531420-000000000-00000. 20642296

[96] Nettis E, Leo ED, Pastore A, Distaso M, Zaza I, Vacca M, Macchia L, Vacca A. *Probiotics and refractory chronic spontaneous urticaria. European Annals Allergy Clin Immunol. 2016;48(5):182–187. 27608474

[97] Bi X-D, Lu B-Z, Pan X-X, Liu S, Wang J-Y. Adjunct therapy with probiotics for chronic urticaria in children: randomised placebo-controlled trial. Allergy Asthma Clin Immunol. 2021;17(1):39. doi:10.1186/s13223-021-00544-3. 33865434 PMC8052813

[98] Yadav MK, Kumari I, Singh B, Sharma KK, Tiwari SK. Probiotics, prebiotics and synbiotics: safe options for next-generation therapeutics. Appl Microbiol Biotechnol. 2022;106(2):505–521. doi:10.1007/s00253-021-11646-8. 35015145 PMC8749913

[99] Arslanoglu S, Moro GE, Boehm G, Wienz F, Stahl B, Bertino E. Early neutral prebiotic oligosaccharide supplementation reduces the incidence of some allergic manifestations in the first 5 years of life. J Biol Regul Homeost Agents. 2012;26(3 Suppl):49–59. 23158515

[100] Kramer MF, Heath MD. Probiotics in the treatment of chronic rhinoconjunctivitis and chronic rhinosinusitis. J Allergy. 2014;2014:1–7. doi:10.1155/2014/983635. PMC4020448 24872820

[101] Atefi N, Fallahpour M, Sharifi S, Ghassemi M, Roohaninasab M, Goodarzi A. Probiotic as an adjuvant therapy in chronic urticaria: a blinded randomized controlled clinical trial. Eur Ann Allergy Clin Immunol. 2022;54(3):123. doi:10.23822/EurAnnACI.1764-1489.200. 33939347

[102] Zhang F, Cui B, He X, Nie Y, Wu K, Fan D. Microbiota transplantation: concept, methodology and strategy for its modernization. Protein Cell. 2018;9(5):462–473. doi:10.1007/s13238-018-0541-8. 29691757 PMC5960466

[103] Jiang X, Liu Z, Ma Y, Miao L, Zhao K, Wang D, Wang M, Ruan H, Xu F, Zhou Q, et al. Fecal microbiota transplantation affects the recovery of AD-skin lesions and enhances gut microbiota homeostasis. Int Immunopharmacol. 2023;118:110005. doi:10.1016/j.intimp.2023.110005. 36924566

[104] Kim J-H, Kim K, Kim W. Gut microbiota restoration through fecal microbiota transplantation: a new atopic dermatitis therapy. Exp Mol Med. 2021;53(5):907–916. doi:10.1038/s12276-021-00627-6. 34017060 PMC8178377

[105] Wu L-Q, Yuan Q-F, Qin Z-C, Xu Y-D, Li L, Xu J-T, He X-X, Xie W-R, Wu L-H. Faecal microbiota transplantation for treatment of chronic urticaria with recurrent abdominal pain and food allergy. Singapore Med J. 2023. doi:10.4103/singaporemedj.SMJ-2021-423.37077050

[106] Leeming ER, Johnson AJ, Spector TD, Le Roy CI. Effect of Diet on the Gut Microbiota: Rethinking Intervention Duration. Nutrients. 2019;11(12):2862. doi:10.3390/nu11122862. 31766592 PMC6950569

[107] Zheng D, Ratiner K, Elinav E. Circadian Influences of Diet on the Microbiome and Immunity. Trends Immunol. 2020;41(6):512–530. doi:10.1016/j.it.2020.04.005. 32359722

[108] Nishida A, Inoue R, Inatomi O, Bamba S, Naito Y, Andoh A. Gut microbiota in the pathogenesis of inflammatory bowel disease. Clin J Gastroenterol. 2018;11(1):1–10. doi:10.1007/s12328-017-0813-5. 29285689

[109] García-Montero C, Fraile-Martínez O, Gómez-Lahoz AM, Pekarek L, Castellanos AJ, Noguerales-Fraguas F, Coca S, Guijarro LG, García-Honduvilla N, Asúnsolo A, et al. Nutritional Components in Western Diet Versus Mediterranean Diet at the Gut Microbiota–Immune System Interplay. Implications for Health and Disease. Implic Health Disease Nutri. 2021;13(2):699. doi:10.3390/nu13020699. PMC7927055 33671569

[110] Lee YH, Kalailingam P, Delcour JA, Fogliano V, Thanabalu T. Olive-Derived Antioxidant Dietary Fiber Modulates Gut Microbiota Composition and Attenuates Atopic Dermatitis Like Inflammation in Mice. Mol Nutr Food Res. 2023;67(10):e2200127. doi:10.1002/mnfr.202200127. 36929605

[111] Sofi F, Cesari F, Abbate R, Gensini GF, Casini A. Adherence to Mediterranean diet and health status: meta-analysis. BMJ. 2008;337(sep11 2):a1344. doi:10.1136/bmj.a1344. 18786971 PMC2533524

[112] Serra-Majem L, Román-Viñas B, Sanchez-Villegas A, Guasch-Ferré M, Corella D, La Vecchia C. Benefits of the Mediterranean diet: Epidemiological and molecular aspects. Mol Aspects Med. 2019;67:1–55. doi:10.1016/j.mam.2019.06.001. 31254553

[113] Ayvaz HH, Kuyumcu A. Effect of the Mediterranean diet in patients with chronic spontaneous urticaria. Rev Assoc Medica Bras 1992. 2021;67(5):675–680. doi:10.1590/1806-9282.20201076. 34550255

[114] Di Daniele N, Noce A, Vidiri MF, Moriconi E, Marrone G, Annicchiarico-Petruzzelli M, D’Urso G, Tesauro M, Rovella V, De Lorenzo A. Impact of Mediterranean diet on metabolic syndrome, cancer and longevity. Oncotarget. 2017;8(5):8947–8979. doi:10.18632/oncotarget.13553. 27894098 PMC5352455

[115] Bifulco M. Mediterranean diet: the missing link between gut microbiota and inflammatory diseases. Eur J Clin Nutr. 2015;69(9):1078. doi:10.1038/ejcn.2015.81. 26014263

[116] Ortega MA, Fraile-Martínez O, Naya I, García-Honduvilla N, Álvarez-Mon M, Buján J, Asúnsolo Á, de la Torre B. Type 2 Diabetes Mellitus Associated with Obesity (Diabesity). The Central Role of Gut Microbiota and Its Translational Applications. Nutrients. 2020;12(9):2749. doi:10.3390/nu12092749. 32917030 PMC7551493

[117] Merra G, Noce A, Marrone G, Cintoni M, Tarsitano MG, Capacci A, De Lorenzo A. Influence of Mediterranean Diet on Human Gut Microbiota. Nutrients. 2020;13(1):7. doi:10.3390/nu13010007. 33375042 PMC7822000

[118] Panda AK, Ravindran B, Das BK. Rheumatoid arthritis patients are free of filarial infection in an area where filariasis is endemic: comment on the article by Pineda et al. Arthritis Rheum. 2013;65(5):1402–1403. doi:10.1002/art.37883. 23400937

[119] Fleming JO. Helminth therapy and multiple sclerosis. Int J Parasitol. 2013;43(3–4):259–274. doi:10.1016/j.ijpara.2012.10.025. 23298637

[120] Wammes LJ, Mpairwe H, Elliott AM, Yazdanbakhsh M. Helminth therapy or elimination: epidemiological, immunological, and clinical considerations. Lancet Infect Dis. 2014;14(11):1150–1162. doi:10.1016/S1473-3099(14)70771-6. 24981042

[121] Wördemann M, Diaz RJ, Heredia LM, Collado Madurga AM, Ruiz Espinosa A, Prado RC, Millan IA, Escobedo A, Rojas Rivero L, Gryseels B, et al. Association of atopy, asthma, allergic rhinoconjunctivitis, atopic dermatitis and intestinal helminth infections in Cuban children. Trop Med Int Health TM IH. 2008;13(2):180–186. doi:10.1111/j.1365-3156.2007.01988.x. 18304263

[122] Croft AM, Bager P, Kumar S. Helminth therapy (worms) for allergic rhinitis. Cochrane Database Syst Rev. 2012;2012:CD009238. doi:10.1002/14651858.CD009238.pub2. 22513973 PMC7388915

[123] Brosschot TP, Reynolds LA. The impact of a helminth-modified microbiome on host immunity. Mucosal Immunol. 2018;11(4):1039–1046. doi:10.1038/s41385-018-0008-5. 29453411

[124] Flohr C, Quinnell RJ, Britton J. Do helminth parasites protect against atopy and allergic disease?. Clin Exp Allergy J Br Soc Allergy Clin Immunol. 2009;39(1):20–32. doi:10.1111/j.1365-2222.2008.03134.x. 19128351

[125] Hu Z, Zhang C, Sifuentes-Dominguez L, Zarek CM, Propheter DC, Kuang Z, Wang Y, Pendse M, Ruhn KA, Hassell B, et al. Small proline-rich protein 2A is a gut bactericidal protein deployed during helminth infection. Science. 2021;374(6568):eabe6723. doi:10.1126/science.abe6723. 34735226 PMC8977106

